# Acute Pyelonephritis With Cullen’s Sign Masquerading As Pancreatitis

**DOI:** 10.7759/cureus.23222

**Published:** 2022-03-16

**Authors:** Gurpremjit Singh, Ankur Mittal, Vikas K Panwar, Rudra Ghorai, Akshaya Upadhyay

**Affiliations:** 1 Urology, All India Institute of Medical Sciences, Rishikesh, Rishikesh, IND

**Keywords:** urinary tract infection dka, kidney infections., retroperitoneal pathology, acute pyelonephritis, cullen's sign

## Abstract

Cullen's sign is well described in the literature as subcutaneous ecchymosis in the periumbilical region. It is most commonly represented with acute pancreatitis. Recently, there have been many case reports associated with this sign to different clinical scenarios. A 61-year-old gentleman reported to the ED with left flank pain, intermittent fever, and a periumbilical ecchymosis on abdominal examination. Numerous tests were performed to rule out the likelihood of acute pancreatitis. The patient was diagnosed with acute pyelonephritis and received appropriate treatment. Cullen's sign should raise suspicions of retroperitoneal or intraabdominal abnormalities. While the pathophysiological process underlying the emergence of this symptom frequently signals retroperitoneal bleed, this is not always the case.

## Introduction

Cullen's sign is well described in the literature as subcutaneous ecchymosis in the periumbilical region. It is most commonly described with acute pancreatitis. Recently, there have been many case reports associated with this sign to different clinical scenarios. [1] It is referred to as a complication of hemorrhagic pancreatitis. The sign is named after Thomas S. Cullen, a Canadian gynecologist who researched gynecological diseases. He described the sign in 1918, following a case of a ruptured extrauterine pregnancy [2]. Acute pyelonephritis, an infection of the renal pelvis and kidney resulting from an ascent of a bacterial pathogen from the bladder through the ureter to the kidneys, is one such clinical diagnosis associated with this sign. Herein we describe a case report of the presentation of Cullen's sign with acute pyelonephritis, the patient's clinical management, and course.

## Case presentation

A 61-year-old gentleman presented to the ED with left flank pain and intermittent fever for five days. The pain was a dull aching in character and associated with nausea and vomiting. He had a history of burning micturition and voiding lower urinary tract symptoms for two years. The patient had a recent double J stent (DJS) inserted within the previous two months in an outside hospital for a left ureteric calculus. The patient had a history of smoking, chronic obstructive pulmonary disease, and hypertension. There was no history of trauma or any anticoagulant therapy.

**Figure 1:**
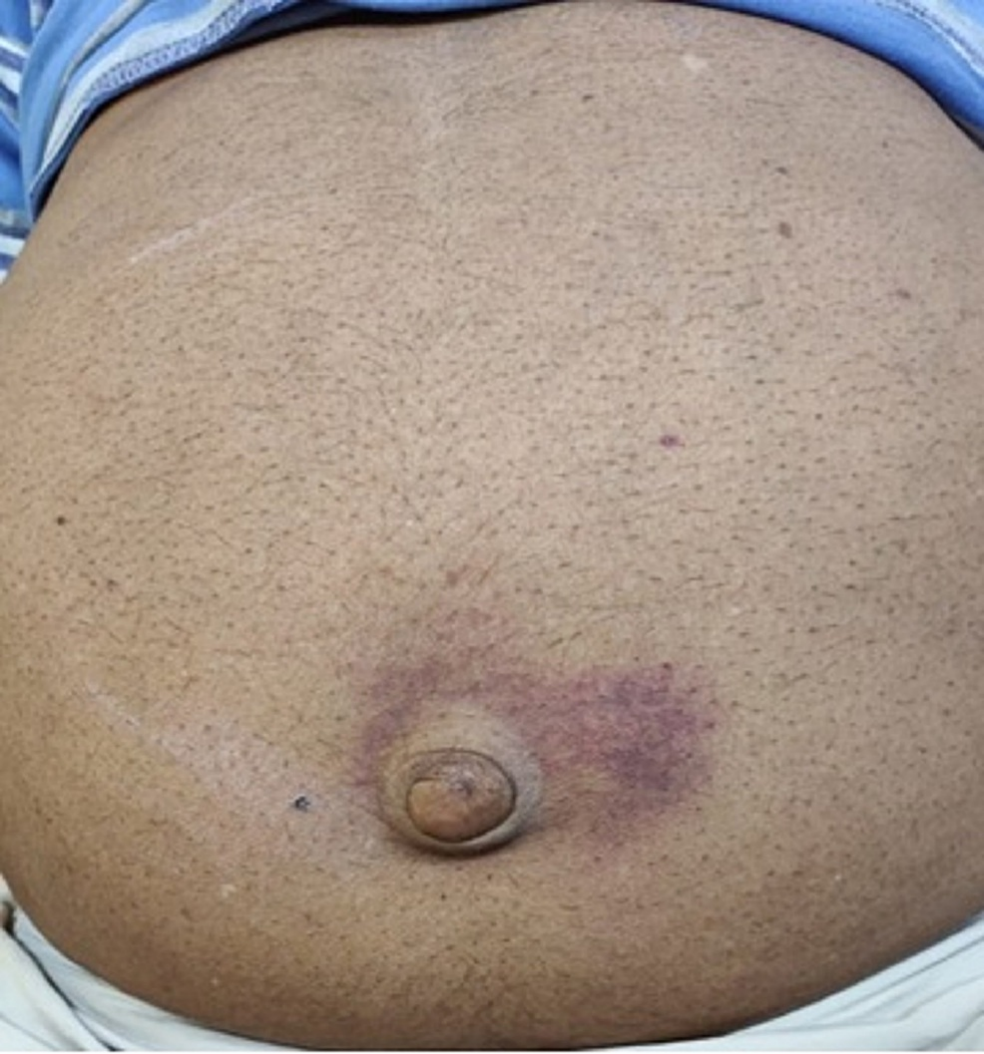
Periumbilical ecchymosis: Cullen's sign.

He had a blood pressure of 140/86 mmHg on general physical examination and a pulse rate of 92/min. His respiratory rate was 18/min, and SpO2 of 97% on room air. Abdominal examination revealed periumbilical ecchymosis (Figure 1) and bilateral (B/L) flank tenderness. The rest of the abdomen was soft non-tender on palpation.

Blood tests are summarised in Table 1. 

**Table 1:** Blood investigations of the patient.

Investigation	Value
Hemoglobin	11.4 g/dL
Total leukocyte count	41330/cubic mm
Urea	68 mg/dL
Creatinine	1.87 mg/dL
Serum amylase	220 IU/L
Serum lipase	98 IU/L

**Figure 2:**
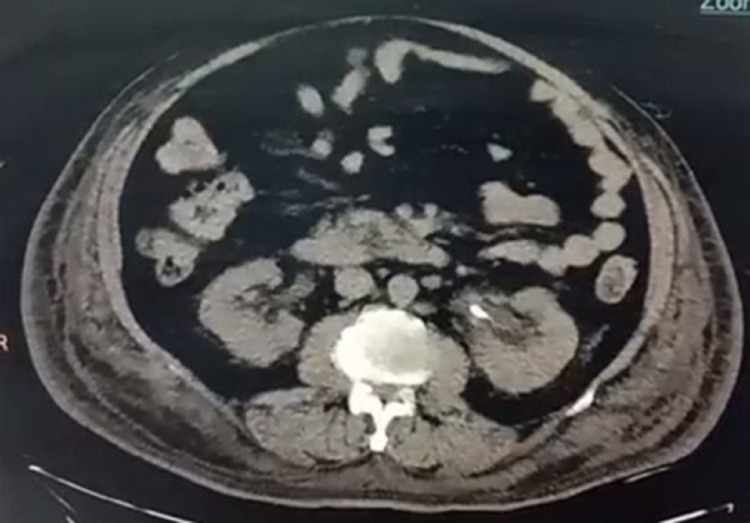
NCCT abdomen showing bilateral perirenal and periureteric fat stranding with left double J stent in situ. NCCT: Non-contrast computed tomography.

**Figure 3:**
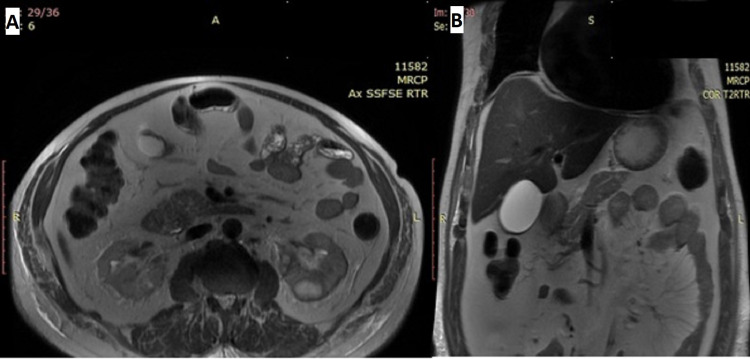
On magnetic resonance cholangiopancreatography, bilateral kidneys show irregular margins with perinephric T2/STIR hyperintense signal. No features suggestive of pancreatitis. (A) Axial section (there is a T1 hypointense cortical cyst in the left kidney at the lower pole), (B) Coronal section. T2/STIR: T2-weighted short-tau inversion recovery.

Ultrasound was performed, which showed few calculi in the gallbladder, largest 8 mm, left renal calculi in upper and mid pole, largest 11 mm, and a left DJS in situ. Non-contrast computed tomography (NCCT) whole abdomen was done, which showed B/L perirenal and periureteric fat stranding with left DJS in situ and B/L pleural effusion (Figure 2). The rest of the organs appeared normal. Magnetic resonance imaging was arranged as the patient displayed elevated amylase and lipase, suggesting pancreatitis, while the pancreas appeared normal on NCCT. The magnetic resonance cholangiopancreatogram showed multiple gallbladder calculi of 5-6 mm. The right and left hepatic ducts, common bile duct, and the pancreatic duct were normal. None of the features were suggestive of pancreatitis. B/L kidneys showed irregular margins with peri-nephric T2-weighted short-tau inversion recovery (STIR) hyperintense signal and a T1 hypointense cortical cyst in the left kidney at the lower pole (Figure 3). The urine culture was sterile, but the blood culture showed Klebsiella pneumoniae sensitive to cefepime and piperacillin-tazobactam.

A per-urethral catheter was placed on admission, and the patient was started on piperacillin-tazobactam and analgesics. The counts gradually decreased on treatment. There were no features to suggest stent blockage as it would have resulted in hydroureteronephrosis. There was steady progress, and he was discharged on day ten on oral antibiotics in hemodynamically stable condition. The oral antibiotics were continued for 14 days. The sign disappeared completely two weeks post-discharge. At six weeks follow-up, the patient was doing well and was comfortable. Left DJS was removed on an outpatient basis, and presently patient is doing well.

## Discussion

Dr. Cullen first described a case report of ruptured ectopic pregnancy with periumbilical discoloration, which later became known as Cullen's sign [3]. It is often described in the association with acute pancreatitis. In their prospective study of 770 patients, Dickson AP and Imrie CW described the overall incidence of 3% of body wall ecchymosis. Only nine patients in this study with pancreatitis had Cullen's sign. Therefore, this sign is neither specific nor sensitive for acute pancreatitis [4].

Cullen's sign has been associated with many clinical scenarios, like an amoebic liver abscess, rectus sheath hematoma, pancreatic trauma, massive ovarian enlargement, perforated duodenal ulcer, splenic injury, non-Hodgkin's lymphoma, perirenal hemorrhage, and as a complication of anticoagulation. The common abnormality in all these cases seems to be retroperitoneal hemorrhage. Chung KM and Chuang SS described the association of perirenal hematoma with Cullen's sign. They described the pathophysiology behind this as blood diffused to the subcutaneous tissue around the umbilicus via falciform ligament. The attachment to the umbilicus is via round ligament of the liver, which forms part of the free edge of the falciform ligament [2,4].

Several theories have been given about the appearance of Cullen's sign. Meyers MA et al., using CT studies, described that blood in the abdominal wall might be the cause of this sign. This blood reaches the periumbilical subcutaneous plane via blood tracking along the round ligament to the umbilicus. The blood from the retroperitoneum goes along the gastrohepatic ligament to the falciform ligament and spreads along the round ligament to the umbilicus. Other theories describe the lymphatic spread of pancreatic enzymes into the subcutaneous tissue resulting in periumbilical ecchymosis [5-7]. Almost all the cases have a retroperitoneal hemorrhage, as the expected finding. The present case report described the association of acute pyelonephritis with the presence of Cullen's sign. This has not been previously described in the literature. In acute pancreatitis, the sign takes 2-3 days to develop and is associated with a mortality of 37% [5,8]. Whereas in the index patient report, the presentation was with Cullen's sign and bilateral flank pain. The association of pancreatitis was ruled out using a CT scan and magnetic resonance cholangiopancreatography. Moreover, the patient had complete recovery following the episode after appropriate management.

On reviewing the literature, it has been found that Cullen's sign has been associated with many secondary conditions in recent years. Cullen's sign should alert the physician to a possibility of retroperitoneal or intraabdominal abnormality. It should be thoroughly investigated using computed tomography. The purpose of this case report is to alert the physician to recognize this sign and the probable differential diagnosis, which should be investigated. Acute pyelonephritis should also be kept as a rare possibility for this sign. Even though the pathophysiological mechanism described in the appearance of this sign suggests a possibility of retroperitoneal blood, it is not always true, even for acute pancreatitis and retroperitoneal hemorrhage [1]. Only further reporting of such cases can help identify the possible etiology of this finding in acute pyelonephritis.

## Conclusions

This report describes the rarity of Cullen's sign and a never known association of this with acute pyelonephritis. Young physicians rarely come across a presentation of Cullen's sign. This case report aims to educate physicians about this symptom and the possible differential diagnosis that should be considered. Cullen's sign may be seen in individuals who have had an ectopic pregnancy rupture, a ruptured aortic aneurysm, or intra-abdominal malignancies, including primary or metastatic liver tumors and acute pyelonephritis. Therefore, this should not be taught to medical students as a sign associated only with acute pancreatitis but with a broad association with different differential diagnoses.

## References

[1] Brkovic D, Moehring K, Doersam J, Pomer S, Kaelble T, Riedasch G, Staehler G (1996). Aetiology, diagnosis and management of spontaneous perirenal haematomas. Eur Urol.

[2] Chung KM, Chuang SS (2011). Cullen and Grey Turner signs in idiopathic perirenal hemorrhage. CMAJ.

[3] Linkletter AM, Moore FC (1961). Cullen's sign in ruptured ectopic pregnancy. Can Med Assoc J.

[4] Dickson AP, Imrie CW (1984). The incidence and prognosis of body wall ecchymosis in acute pancreatitis. Surg Gynecol Obstet.

[5] Harris S, Naina HV (2008). Cullen's sign revisited. Am J Med.

[6] Mabin TA, Gelfand M (1974). Cullen's sign, a feature in liver disease. Br Med J.

[7] Meyers MA, Feldberg MA, Oliphant M (1989). Grey Turner's sign and Cullen's sign in acute pancreatitis. Gastrointest Radiol.

[8] Rahbour G, Ullah MR, Yassin N, Thomas GP (2012). Cullen's sign - Case report with a review of the literature. Int J Surg Case Rep.

